# Preparation of Hierarchical Porous Silicalite-1 Encapsulated Ag NPs and Its Catalytic Performance for 4-Nitrophenol Reduction

**DOI:** 10.1186/s11671-018-2579-1

**Published:** 2018-06-07

**Authors:** Bin Wang, Haojiang Wang, Fengwei Zhang, Tijian Sun

**Affiliations:** 1 Department of Chemistry, Shanxi Medical University, Taiyuan, 030001 People’s Republic of China; 2 Institute of Crystalline Materials, Shanxi University, Taiyuan, 030006 People’s Republic of China

**Keywords:** Hierarchical porous silicalite-1, In situ encapsulation, Silver nanoparticles, 4-nitrophenol, Hydrogenation

## Abstract

A facile and efficient strategy is presented for the encapsulation of Ag NPs within hierarchical porous silicalite-1. The physicochemical properties of the resultant catalyst are characterized by TEM, XRD, FTIR, and N_2_ adsorption-desorption analytical techniques. It turns out that the Ag NPs are well distributed in MFI zeolite framework, which possesses hierarchical porous characteristics (1.75, 3.96 nm), and the specific surface area is as high as 243 m^2^ · g^−1^. More importantly, such catalyst can rapidly transform the 4-nitrophenol to 4-aminophenol in aqueous solution at room temperature, and a quantitative conversion is also obtained after being reused 10 times. The reasons can be attributed to the fast mass transfer, large surface area, and spatial confinement effect of the advanced support.

## Background

The stability of the metal nanoparticles (MNPs) is of great academic and practical importance in heterocatalysis fields because the catalytic activity and selectivity are directly related to the morphology and size of MNPs [1–7]. But it is a pity that the MNPs are usually inclined to aggregate/leaching at relatively high temperatures or during the reaction process, thus leading to a remarkable decrease of catalytic performance [8–11]. To this end, numerous efforts have been devoted to resolving the stability issue of MNPs in heterogeneous catalytic system [12–17]. Conventionally, the surfactant-stabilized MNPs are primarily prepared, and then a layer of porous inorganic/polymeric materials are coated on their surface to improve the MNPs’ stability. On the other hand, the MNPs are dispersed on the solid supports in advance, and then another porous coating is grown on their surface. Although these strategies can effectively enhance the stability of MNPs, the preparation process of the MNP-based catalyst is relatively cumbersome and the MNP surface wrapped surfactant is not conducive for the exposure of active sites.

Over the past decades, the most commonly used catalysts for the chemical reactions depended heavily on supported precious metals, such as Pd, Au, and Pt [18–21]. To a certain extent, they exhibited excellent catalytic performance in a variety of reaction systems, but the limited availability, expensive price, and moderate stability of these noble metals have greatly suppressed their extensive application. In contrast, the extremely low-cost and earth-abundant alternatives, such as Co, Ni, and Cu metals, have displayed great application potential and development prospect [22–28]. Nevertheless, the catalytic performance and stability of the non-noble metal catalysts are suffering from the problems of agglomeration and deactivation. To address these problems, a large number of the current studies have been exploited to encapsulate different kinds of MNPs and to improve their dispersity and stability, but their preparation procedures are still excessively tedious and inefficient [29–35]. Therefore, developing a simple and efficient strategy to prepare MNPs encapsulated catalyst with good dispersibility and stability is urgently desirable.

Based on the above-mentioned reasons, herein, we report a novel one-pot method for the ingenious preparation of Ag NPs@silicalite-1 hybrid catalyst with Ag NPs encapsulated into the hierarchical porous silicalite-1 microspheres (Ag@HPS-1). It is worth mentioning that the high surface area and hierarchical porous characteristics of Ag@HPS-1 catalyst endow special catalytic property and stability of Ag NPs for the reduction of 4-nitrophenol in aqueous solution. In addition, it can be envisioned that after the introduction of other types of single or bimetallic components, more interesting reactions will be found in the future.

## Methods

### Preparation of Hierarchical Porous Silicalite-1 Encapsulated Ag NPs

The hierarchical porous silicalite-1 encapsulated Ag NPs (Ag@HPS-1) catalyst was synthesized according to the following procedure. Firstly, 5.0 g of 40 wt% Ludox HS-40 colloidal silica was added into 20 mL of deionized water and stirred for 0.5 h. The solution pH value was adjusted to 12 by adding a certain amount of 25 wt% NH_3_·H_2_O. Afterwards, a required amount of AgNO_3_ solution was added dropwise. The suspension was continued to be stirred at 80 °C overnight for the complete removal of water. Secondly, 2.0 g of tetrapropyl ammonium hydroxide (TPAOH; 25 wt% in deionized water) was added into the resulting solid powder, then the mixture was transferred into a 25 mL Teflon-lined autoclave for crystallization at 120 °C for 48 h. Finally, the as-prepared solid was heated to 550 °C in air for 8 h to remove all possible organic components and then reduced in 5 vol% H_2_/Ar at 400 °C for 6 h. The HPS-1 support was prepared using the identical procedure except that no AgNO_3_ was added during the reaction course.

### Catalytic Reduction of 4-Nitrophenol by Ag@HPS-1 Catalyst

Typically, the mixture of Ag@HPS-1 catalyst aqueous suspension (20 mL, 0.8 g/L), 4-nitrophenol (5 mL, 3 mM), and NaBH_4_ aqueous solution (5 mL, 0.3 M) was stirred at room temperature. After a period of reaction, the upper solution was transferred to a quartz cuvette for ultraviolet visible spectroscopy (UV-Vis) measurement. Once a spectrum was acquired, the solution was immediately transferred back to the reaction vessel, and stirring was continued for the sequential reduction reaction until the bright yellow gradually turned colorless. To study the catalyst’s recyclability, the catalyst was separated by centrifugation after each reaction was completed. Thereafter, the catalyst was washed three times with deionized water and ethanol and used for the subsequent recycle under the same reaction conditions.

### Characterization of Phisicochemical and Catalytic Properties

Transmission electron microscope (TEM) was carried out on a Tecnai G2F30 using an accelerating voltage of 200 kV. The samples were obtained by placing a drop of colloid solution onto a micro grid and evaporated in air under infrared light irradiation. XRD measurements were performed on a Rigaku D/max-2400 diffractometer using Cu-Kα radiation as the X-ray source in the 2θ range of 10^o^–90^o^. The N_2_ adsorption-desorption isotherms were achieved on an ASAP2020 analyzer. Before measurement, the samples were degassed under vacuum at 393 K for 8 h. Specific surface area of the samples was calculated by the Brunauer-Emmet-Teller (BET) method, pore volume, and pore size distribution were calculated applying the Barrett-Joyner-Halenda (BJH) model. Fourier transform infrared (FTIR) spectra were obtained using a NEXUS 670 spectrophotometer (frequency range from 4000 to 400 cm^−1^) with KBr pellet. Ultraviolet visible spectroscopy was conducted with a UV2800PC UV-vis spectrophotometer.

## Results and Discussion

The highly active and stable Ag@HPS-1 catalyst was prepared according to the Yu’s group and with some modifications [36]. The catalyst was firstly prepared via a facile one-pot method, where the commercial colloidal silica and silver nitrate were used as silicalite and metal species precursors, respectively. And the obtained Ag NPs were all encapsulated into the hierarchical porous silicalite-1 microspheres, which was well consistent with the literature reported results. Briefly, during the formation of large silica-based assembly in alkaline medium, the positively charged Ag(NH_3_)_2_^+^ in the solution was firmly binded to the negatively charged silica aggregates via the electrostatic self-assembly to form stable nanocomposites. Subsequently, the produced sample was treated under hydrothermal and high temperature treatment conditions. The loading amount of Ag on the catalyst was measured to be about 2.96 wt%.

**Fig. 1:**
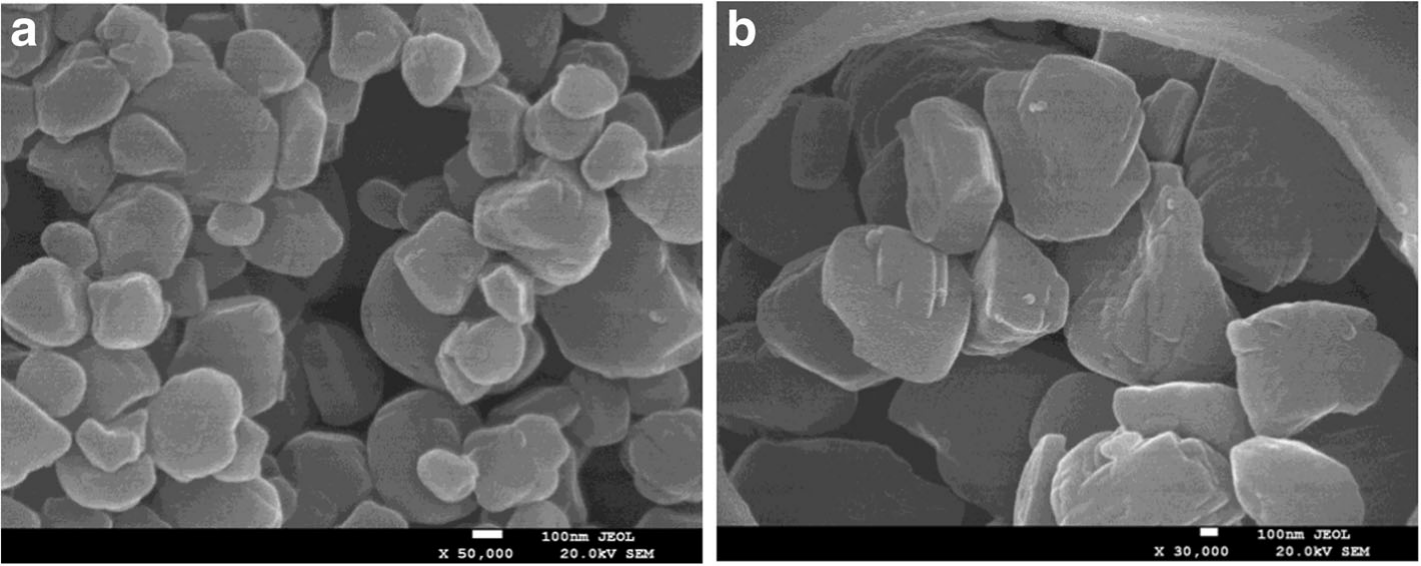
**a** SEM image of HPS-1 support and **b** Ag@HPS-1 catalyst

**Fig. 2:**
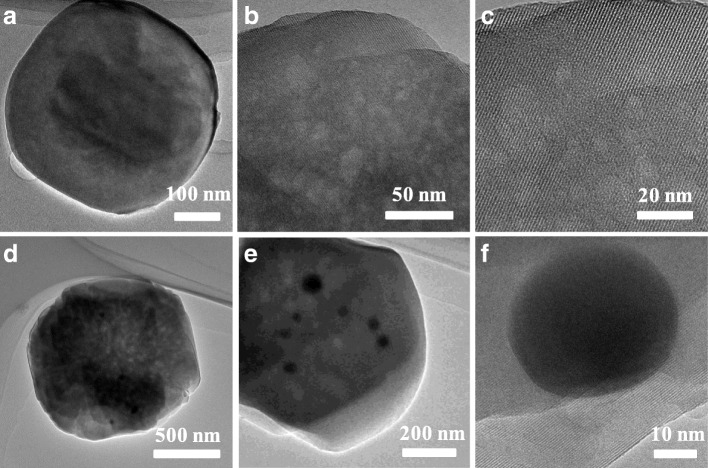
**a–c** The TEM and HRTEM images of HPS-1. **d–f** The TEM and HRTEM images of Ag@HPS-1 catalyst

The morphology and size of HPS-1 and Ag@HPS-1 samples were observed through scanning and transmission electron microscopy (SEM, TEM), shown in Figs. 1 and 2. The low-resolution SEM images of HPS-1 and Ag@HPS-1 samples were revealed that the HPS-1 support has a relatively uniform shape and size, while the Ag@HPS-1 catalyst has a larger size and the Ag NPs cannot be observed. From the TEM image, it can be clearly seen that the as-prepared silicalite zeolite nanocrystals were close to spherical in shape with an average particle size around 455 nm. The HRTEM image of HPS-1 microsphere, with clearly distinguishable and oriented lattice fringes of MFI zeolite, suggested the high crystallinity of the synthesized silicalite-1 nanocrystal. In addition, there were also a number of disordered tiny holes on the nanocrystal surface, revealing the characteristic of hierarchical porosity (Fig. 2a–c). Interestingly, all of the diameter ca. 25-nm Ag NPs were in situ encapsulated in Ag@HPS-1 and reserved the morphology of HPS-1 except that the particle size was increased to 1.25 μm (Fig. 2d–f).

**Fig. 3:**
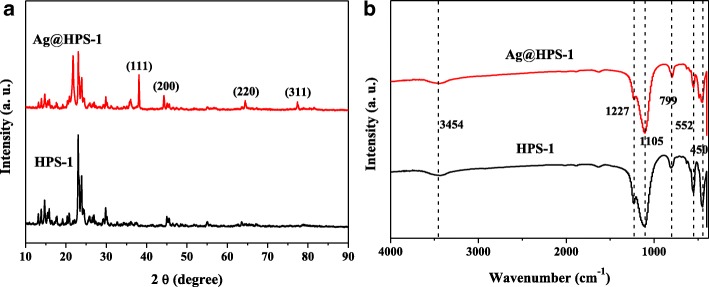
**a** XRD patterns. **b** FTIR spectra of the as-prepared HPS-1 and Ag@HPS-1 samples

The degree of crystallinity of the as-prepared samples was further determined by X-ray powder diffraction (XRD). The XRD patterns of HPS-1 and Ag@HPS-1 were shown in Fig. 3a. As depicted in Fig. 3a, both of them exhibited the characteristic peaks of MFI structure, indicating the formation of silicalite-1, which are consistent with the corresponding TEM results [37]. Besides that, the Ag@HPS-1 catalyst exhibited another four characteristic diffraction peaks at 2θ = 38.1°, 44.3°, 64.4°, and 77.4°, corresponding to the (111), (200), (220), and (311) planes of face-centered cubic (fcc) structure of Ag NPs [38, 39]. The XRD results confirming the metallic Ag NPs have been successfully generated after reduction treatment with 5 vol% H_2_/Ar at 400 °C. The FTIR spectroscopy was employed to determine the possible organic functional groups of the as-prepared HPS-1 and Ag@HPS-1 samples. As shown in Fig. 3b, the relatively weak and broad peaks at 1636 and 3454 cm^−1^ could be assigned to the surface-adsorbed water molecules, O–H stretching and symmetric bending vibrations. The absorption bands at around 1105 and 799 cm^− 1^ were corresponded to the antisymmetric and symmetric stretching vibrations of Si–O–Si for HPS-1 support. In comparison with HPS-1, the characteristic absorption peaks had almost no change after the encapsulation of Ag NPs within the HPS-1 microsphere, indicating that the nitrogen-containing species have been escaped from the material’s surface during the heat treatment.

**Fig. 4:**
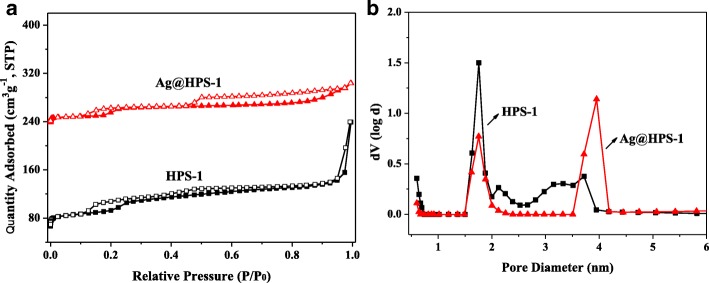
**a** The N_2_ adsorption-desorption isotherms and **b** the corresponding pore size distribution curves of HPS-1 and Ag@HPS-1

N_2_ adsorption-desorption isotherms and the corresponding pore size distributions of HPS-1 and Ag@HPS-1 were presented in Fig. 4. Both of these two samples can be classified as type IV isotherms and with small hysteresis loop (Table 1). The specific surface area and total pore volume of HPS-1 were calculated to be 413 m^2^ · g^−1^, 0.394 cm^3^ · g^−1^, while it possesses 1.76 and 3.67 nm two kinds of channel model. For Ag@HPS-1 catalyst, it was reserved the hierarchical porous characteristics (1.75, 3.96 nm), while the surface area and total pore volume was just slightly decreased to 243 m^2^ · g^−1^ and 0.176 cm^3^ · g^−1^. The reasons for the reduced specific surface area and pore volume are presumably because the Ag NPs were well embedded into the framework of silicalite-1. It is well acknowledged that the microporous structure will help the substrates to be concentrated inside the pores and then reduced on the surface of Ag NPs. Moreover, the mesoporous characteristic is in favor of the efficient transport of substrate and product. From the above results, it can be speculated that the high surface area and hierarchical pore of Ag@HPS-1 will obviously improve the mass transfer rate of reaction substrates and the stability of heterogeneous catalyst.

**Table 1:** The results of N_2_ adsorption-desorption for different zeolite-based samples

Sample	*S*_BET_ (m^2^ · g^−1^)^a^	*V*_total_ (cm^3^ · g^−1^)^b^	Pore size (nm)^c^	Reference
HPS-1	413	0.394	1.76, 3.67	This work
Ag@HPS-1	243	0.176	1.75, 3.96	This work
Pd@mnc-S1	338	0.35	0.45, 3.0	[8]
Pd/S-1-in	378	–	0.53	[36]
Pd@S-1	371	0.15	0.55	[37]
CuHS-1R	247	0.387	6.2	[45]

^a^*S*_BET_ is calculated using BET method

^b^*V*_total_ is the single point adsorption at *P*/*P*_0_ = 0.99

^c^Average pore diameter, pore size is calculated using HK or BJH method

**Fig. 5:**
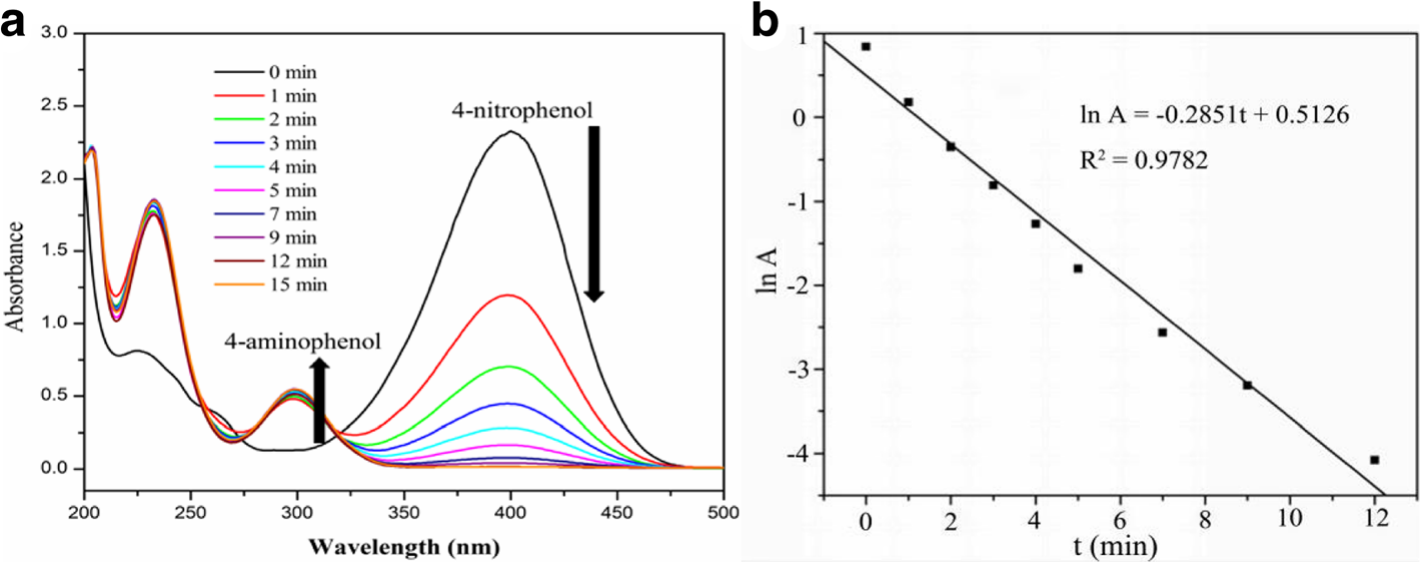
**a** The time-dependent UV-vis absorption spectrum and **b** plot of lnA versus time for the reduction of 4-NP to 4-AP in the presence of Ag@HPS-1 catalyst

The catalytic performance of the homemade Ag@HPS-1 was evaluated using the reduction of 4-nitrophenol (4-NP) to 4-aminophenol (4-AP) as a model catalytic reaction. Figure 5a displays the time-dependent UV-vis absorption spectra of the conversion of 4-NP in the presence of Ag@HPS-1 catalyst in a batch reactor system. After adding NaBH_4_ solution into the above suspension, the color of the solution immediately changed from light yellow to bright yellow owing to the production of 4-nitrophenolate ions. The intensity of the characteristic absorption peak at 400 nm associated with 4-nitrophenolate ions gradually decreased with the reaction time because of the continuous consumption of them. Meanwhile, a new absorption peak at 300 nm associated with product emerged and increased successively with time. Such reaction could be completed within 20 min at room temperature, which could be detected by direct observing the color of the aqueous solution (the color changed from bright yellow to colorless). Furthermore, a plot of lnA versus time allowed to obtain information about the kinetics of the reaction (Fig. 5b). Given that the reduction was conducted in the presence of a large excess of NaBH_4_, the reaction rate is independent of NaBH_4_ concentration and could be regarded as a pseudo-first-order with respect to 4-NP. The apparent rate constant (*k*) was determined from the slope to be 4.75 × 10^−3^ s^−1^ at 298 K, which was comparable to or even higher than the literature reported values, such as the bare Ag NPs (2.1 × 10^−3^ s^−1^), Ag@HTO-PDA catalyst (3.14 × 10^−3^ s^−1^), Ag NPs@PGMA-SH (3.94 × 10^−3^ s^−1^), CNC/CTAB supported Ag NPs (5.76 × 10^−3^ s^−1^), carbon nanofibers/silver nanoparticles (6.2 × 10^−3^ s^−1^), and MWCNTs-polymer-supported AgNPs (7.88 × 10^−3^ s^−1^) [38, 40–44]. The results demonstrated that the reduction of 4-NP to 4-AP could be effectively catalyzed in the presence of Ag@HPS-1catalyst.

**Fig. 6:**
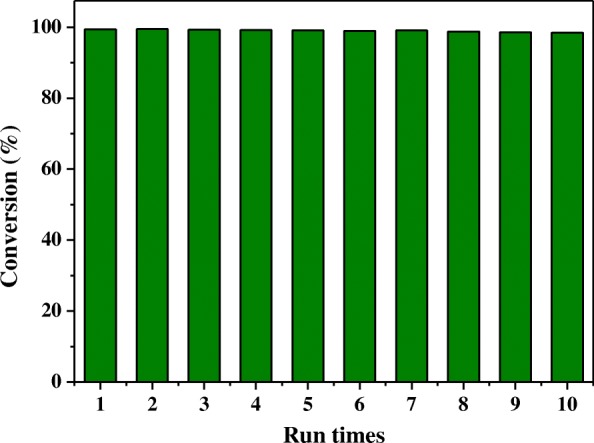
Recycling tests for the reduction of 4-nitrophenol over the Ag@HPS-1 catalyst

The recyclability and stability for reduction of 4-nitrophenol over Ag@HPS-1 catalyst was also investigated as the reusability of the heterogeneous catalyst is one of the most important issues for practical applications. After completion of the previous run, the catalyst was re-collected and dried for the next cycle without reduction again. As shown in Fig. 6, the catalytic activity was almost the same as that obtained with the fresh prepared catalyst after six cycles. It should be noted that the catalyst gave an over 98% conversion within 20 min under each cycle. Most importantly, after the catalytic reaction, the size of Ag NPs as well as the morphology and crystallinity of the zeolite catalyst remained unchanged, demonstrating that the catalyst of Ag NPs encapsulated within HPS-1 microspheres had superior recyclability during the reaction process.

## Conclusions

In conclusion, a facile and in situ encapsulation strategy has been successfully developed to synthesize uniform Ag NPs embedded within the hierarchical porous zeolite using colloidal silica and silver nitrate as silicalite precursor and metal active species. The as-prepared catalyst showed superior thermal, reusability stability as well as excellent catalytic activity for reduction of 4-nitrophenol owing to the confinement of Ag NPs within the zeolite matrix, high surface area, and hierarchical porous characteristics. Furthermore, the present Ag-based catalyst could be recovered in a facile manner from the reaction mixture and without any significant loss in its activity even after 10 cycles. It can also be envisioned that after introducing other types of single or bimetallic components, more interesting reactions will be found in the future.

## Abbreviations

4-AP: 4-Aminophenol
4-NP: 4-Nitrophenol
Ag NPs: Silver nanoparticles
Ag@HPS-1: Hierarchical porous silicalite-1 encapsulated Ag NPs
BET: Brunauer-Emmet-Teller method
BJH: Barrett-Joyner-Halenda model
CNC: Cellulose nanocrystal
CTAB: Hexadecyltrimethylammonium
FTIR: Fourier transform infrared spectrum
HPS: Hierarchical porous silicalite-1 microspheres
HTO-PDA: Hollow-tubular-oriented polydopamine
MNPs: Metal nanoparticles
MWCNTs: Multiwalled carbon nanotubes
PGMA-SH: Sulfhydryl-functionalized poly(glycidyl methacrylate) microspheres
TEM: Transmission electron microscope
TPAOH: Tetrapropyl ammonium hydroxide
UV-vis: Ultraviolet visible spectroscopy
XRD: X-ray powder diffraction
